# Method development to characterise elephant tail hairs by LA-ICP-MS to reflect changes in elemental chemistry

**DOI:** 10.1007/s10653-022-01207-x

**Published:** 2022-02-07

**Authors:** Fiona Sach, Lorraine Fields, Simon Chenery, Lisa Yon, Michelle D. Henley, Peter Buss, Ellen S. Dierenfeld, Simon C. Langley-Evans, Michael J. Watts

**Affiliations:** 1 Inorganic Geochemistry, Centre for Environmental Geochemistry, British Geological Survey, Nottingham, UK; 2 School of Biosciences, University of Nottingham, Nottingham, UK; 3 School of Veterinary Medicine and Science, University of Nottingham, Nottingham, UK; 4 Applied Behavioural Ecology and Environmental Research Unit, University of South Africa, Pretoria, South Africa; 5 Elephants Alive, Bosbokrand, Limpopo South Africa; 6 Veterinary Wildlife Services, South African National Parks, Kimberley, South Africa; 7 LLC, Saint Louis, MO 63128 USA; 8 School of Animal, Rural & Environmental Sciences, Nottingham Trent University, Nottingham, UK

**Keywords:** Environmental geochemistry, Minerals, Potentially toxic elements, Spatial analysis, Bio-indicator, Elephant

## Abstract

This paper evaluated analytical methods used to generate time-series data from elephant tail hairs, which can be used to reflect changing exposure to environmental geochemistry. Elephant tail hairs were analysed by three methods sequentially, each providing data to inform subsequent analysis. Scanning Electron Microscopy (SEM) and X-ray Microanalysis visually showed the structure of the hair, specific structures such as tubules, and the mineral crusting around the edge of the hair, informing targeting of subsequent analysis by Laser Ablation-Inductively Coupled Plasma Mass Spectrometry (LA-ICP-MS). LA-ICP-MS generated time-series data which informed sectioning of the tail hairs for subsequent quantitative analysis for potentially toxic elements and micronutrients using Inductively Coupled Plasma Mass Spectrometry (ICP-MS) of dissolved tail hairs. This novel approach to characterise the tail hair enabled time-series analysis to reflect changes in environmental exposure which may result from seasonal or geochemical spatial variation and could inform elephant movement patterns. The seasonal change between wet and dry seasons was reflected down the length of the hair. Correlations were seen between LA-ICP-MS data and ICP-MS data in several elements including Mg, P, Ca, Fe, Na, Mn and U. This study provided time-series data for the analysis of elephant tail hairs by evaluating analytical challenges to obtaining quantitative data, such as improving protocols to ensure removal of extraneous material, determining where to section the tail hairs to best reflect environmental changes/exposure and ensuring representative analyses. A protocol was established to determine mineral status across a 12–18 month time period utilizing single elephant tail hairs.

**Supplementary Information:**

The online version contains supplementary material available at 10.1007/s10653-022-01207-x.

## Introduction

The structure of elephant tail hair in relation to elemental distribution across and down the hair is largely unknown. Previous work indicated that the structure of tail hair is similar to hair from other mammalian species (Hausman, 1920; Yates et al., 2010). In human and horse hair, relative concentrations of certain biologically required elements or potentially toxic elements (PTEs), varied down the hair, reflecting changes in dietary intake over time (Armelin et al., 2003; Bencko, 1995; Combs, 1987; Middleton et al., 2016). Sach, Dierenfeld, et al. (2020) demonstrated that elephant tail hair is a reliable indicator of Fe and As intake and thus proxy for status, in African (*Loxodonta africana)* and Asian elephants *(Elephas maximus)* in UK zoos. Given an approximate growth rate for a typical wild elephant hair of 0.56 mm/day (± 0.11) in males and 0.81 mm/day (± 0.13) in females, based on two studies of 32 and 50 elephant hairs, respectively, (Cerling et al., 2004; Wittemyer et al., 2009) an elephant hair of 30 cm length has the potential to indicate mineral and PTE status over a 12–18 month time period.

A non-invasive bio-indicator to determine changes in elemental chemistry could be used to reflect mineral/micronutrient status or PTE exposure of an elephant, with the potential to inform spatial movements in obtaining food and other resources, particularly from contrasting environments (Chiyo et al., 2005; Sach, Yon, et al., 2020). Generally, plants will reflect the soil mineral profile, and those growing in mineral deficient areas will lack key minerals, thus potentially resulting in deficiencies of supply to the consumer (Hurst et al., 2013; Joy et al., 2015). Correlation of seasonal elephant food intake to tail hair samples was demonstrated by Cerling et al. (2004) with two elephants’ tail hairs from Tsavo, Kenya, using stable carbon isotope analysis to reflect the seasonal changes in diet, leaves versus grass. Additionally, the difference between dry and wet season diets was further demonstrated by Wittemyer et al. (2009) using similar stable isotope analysis techniques on 50 elephant tail hairs from 18 elephants in Kenya.

There is limited literature on using tail hair time-series analysis to reflect variation in an elephant’s mineral and PTE status, and the correlation between tail hair mineral levels and an animal’s dietary intake. Laser Ablation-Inductively Coupled Plasma Mass Spectrometry (LA-ICP-MS) is a non-destructive technique frequently employed to provide time-series elemental data from samples requiring a temporal profile. For example, this can be used in fish otoliths (ear bones), for which microspatial analysis across growth rings represents a time and geochemical profile relating to the movement patterns of fish populations (Hoover & Jones, 2013), and exposure to geochemical variations (Milton & Chenery, 2005). In this study, LA-ICP-MS measurement of elephant tail hair was used as a non-destructive technique to reflect changes in elemental chemistry, thereby inferring changes in geochemical exposure that may result from temporal movement patterns, changes in habitat or seasonal changes that influence dietary intake, and therefore exposure to environmental minerals or PTEs.

The aim of this study was to develop an analytical method to reflect changes in exposure to differing geochemistry using elephant tail hair samples. This aim was pursued through the following objectives:

1. Develop protocol to inform LA-ICP-MS microspatial analysis for an elephant tail hair using SEM to account for potential sample inhomogeneity and location for micro-sampling transects.
2. Generate rapid microspatial chemistry data using LA-ICP-MS with a time profile down the length of an elephant tail hair—confirm with solution chemistry-ICP-MS high and low concentration points reflecting contrasting environments.

## Methods and materials

### Sample collection

Elephant tail hairs used for this investigation were collected from African elephants (*Loxodonta Africana*) on the Palabora Mining Company (PMC) land near Phalaborwa town, South Africa. Tail hair samples were plucked from the tail (1–3 hairs per animal) of seven elephants immobilised for routine collaring operations or management activities by Elephants Alive (2017). This study utilised one sample from a male elephant for elemental mapping, and two further samples from one male and one female, for laser ablation studies and the remaining samples for imaging work. Immobilization was performed according to the South African National Parks Standard Operating Procedures (SANParks SOPs) for Capture Transport (*Standard Operating Procedures for Capture Transport and maintenance in Holding Facilities of Wildlife*, 2017).

Collection and import of samples were covered by the following permits:

(i) United Kingdom ‘Department for Environment, Food and Rural Affairs (DEFRA) authorisation for the importation from third countries of research and diagnostic samples’, ITMP17.0821B—tail hair samples.
(ii) Convention on international trade in endangered species (CITES) of wild fauna and flora for export from South Africa and import into the UK Export 171,485/Export 222,457, Import 566,134/01/Import 568,473/01

Additionally, samples from UK zoo elephants (*n* = 2) were used in method development, and for further examination of the structure of the hair; collected as per methods described in Sach, Dierenfeld, et al. (2020).

### Sample preparation

Prior to analysis, tail hair samples were cleaned by physical removal of extraneous material before using a series of acetone and deionised water rinsing/sonication steps repeated three times, based on the method described by Button et al. (2009). Analytical methods used to examine the tail hairs were: (1) Scanning Electron Microscopy and X-ray Microanalysis to inform appropriate set-up of; (2) Laser Ablation-Inductively Coupled Plasma Mass Spectrometry (LA-ICP-MS) used to determine geochemical patterns and where to section the tail for solution chemistry by; (3) Inductively Coupled Mass Spectrometry (ICP-MS). The same tail hairs were used for LA-ICP-MS and ICP-MS; analysis was conducted sequentially.

To assess the variance in elemental profiles down elephant hairs (representing changes over time), hairs were sectioned horizontally for ICP-MS analysis. The minimum mass of sample for ICP-MS is approximately 0.03–0.05 g in order to maintain data reproducibility. This equated to 3–5 cm of tail hair, depending on hair thickness. Information was needed about the overall structure and morphology of the elephant hair, to inform where to direct the laser for LA-ICP-MS and the appropriate sampling depth, width and laser cleaning protocol. Scanning electron microscopy (SEM) was identified as the optimum technique for this purpose, to examine the structure cross-sectionally and suggest the most appropriate location to direct the laser to avoid mineralised deposits, or depressions from which it can be difficult to remove extraneous material to avoid potential contamination. The LA-ICP-MS data were to be qualitative (distinguishing high/low concentration zones), due to a lack of suitable standards. Subsequent solution ICP-MS analysis was used to provide quantitative data, using dissolved tail hairs—development of appropriate LA-ICP-MS standards would avoid the need for solution ICP-MS and preserve the sample for later use. After the LA-ICP-MS had been conducted, additional examination was undertaken using a Nikon SNZ 1500 binocular zoom microscope with Nikon NIS elements D camera software with a D5-Fi1 camera to assess the optimum depth of laser sampling to provide sufficient/measurable ablated material to the ICP-MS.

To estimate the seasonal time period from which a sample originated, the approximate growth rate of an elephant tail hair, reported in the literature (Cerling et al., 2004; Wittemyer et al., 2009) was used in conjunction with the recorded date on which the tail hair samples were collected. Growth rate must always be considered as an estimation, there are a number of variables which may affect this rate, including when the hair started growing, and whether or not the growth rate was constant.

#### Scanning electron microscopy and X-ray microanalysis method

Sub-samples of the elephant hairs were mounted both longitudinally and as cross-sections using carbon tape placed on a petrographic glass slide and examined in the uncoated state using scanning electron microscopy (SEM) using both secondary electron (SE) imaging and backscattered scanning electron microscopy (BSEM) electron imaging. Elemental distributions in the elephant hairs were studied using digital energy-dispersive X-ray microanalysis (EDXA) elemental mapping and quantitative energy-dispersive electron probe point microanalysis (ED-EPMA). The sub-samples were examined by BSEM imaging using the SEM instrument in the low-vacuum environmental mode to gain a rapid initial understanding of the effects of laser ablation processes on the elephant tail hair to guide the focus of more detailed observations.

BSEM-EDXA analyses were carried out using a QUANTA600 from FEI (ThermoFisher Scientific) environmental scanning electron microscope (ESEM) fitted with a 2-element (diode-type) backscattered electron detector and equipped with an Oxford Instruments INCA 450 energy-dispersive X-ray microanalysis (EDXA) system with a 50 MM^2^ Peltier-cooled (liquid nitrogen free) silicon drift X-ray detector capable of operating at very high input X-ray count rates (up to ~ 10^6^ counts per second). The scanning electron microscope was operated in low vacuum mode (0.95 torr) using a 20 kV electron beam accelerating potential, a beam current of ~ 0.6 nA, and a working distance of 10 mm. Phase identification was aided by microchemical information obtained from observation of semi-quantitative EDXA spectra recorded from features of interest.

Digital EDXA X-ray element maps were recorded for key areas at a resolution of 1024 × 1024 pixels, and using a 20 kV electron beam, ~ 0.6 nA beam currents and at a working distance of 10 mm, to give optimum X-ray count rates of up to 28,000 counts per second. EDXA spectra and digital X-ray elemental maps were processed using the INCA Microanalysis Suite version 5.05 (Oxford Instruments Analytical Limited, 2014) software package. X-ray element maps were produced by summation of data recorded from multiple frame scans to produce maps with sufficient X-ray counts per pixel to enable the key elements, required for the differentiation of the mineral species present, to be detected above background noise (typically between 20 and 50 frame scans, recorded over 0.5–2 h). A reference BSE image was recorded for each mapped area. The image brightness in BSE images is related to the average atomic number of the phases observed, thereby enabling the differentiation of the minerals present. X-ray elemental maps were processed to show relative element concentrations using a ‘rainbow/gradient colour scale’ ranging from black (representing zero background) through to green, yellow and orange (low to intermediate concentration) to red or white (representing high concentration).

#### Laser ablation-inductively coupled plasma mass spectrometry

Previous work indicated that LA-ICP-MS was a useful tool for trace element characterisation in a single hair (Sela et al., 2007). For the elephant hairs, elements of interest were examined both longitudinally down the hair and cross-sectionally: Na, Mg, P, K, Ca, Mn, Zn, Pb and U. Longitudinal examination was of principal interest to determine if elemental levels reflected significant changes down the hair, and therefore, if changes over time could be identified. After method development trials, LA-ICP-MS was conducted on hairs from two animals. Cross-sectional LA-ICP-MS was also conducted to assess elemental change across the hair to ensure the depth of longitudinal LA-ICP-MS sampling was appropriate.

Tail hairs were fixed to the laser plate using double-sided adhesive tape. Hairs were coiled around a glass reference material, SRM 610 (NIST, USA), that was used to ensure appropriate calibration of the laser. The ablation was conducted with a NewWave UP193FX excimer (193 nm) laser system, with built-in microscope imaging, which was coupled to an Agilent 7500 series ICP-MS, with helium gas, mixed with argon. Laser conditions such as energy and beam size were determined from preliminary ablations on spare hairs; energy was 20 Hz at 100% power. The irradiance was typically 1.51 GW/cm^2^ and fluence was 7.54 J/cm^2^. Laser ablation crater diameters were set at 100 μm. To remove any extraneous surface material, two cleaning passes were run over the hair followed by one single data collection pass. The cleaning speed was 200 microns/sec (2 passes) followed by a scanning speed of 100 microns/sec (1 pass), this being set using the UP193nm software. Laser Ablation ICP-MS operating parameters are given in Supplementary Table 3.

#### Inductively coupled plasma mass spectrometry (ICP-MS) analysis of dissolved tail hair samples

Sectioned elephant tail hair samples were also digested using a MARS Express closed vessel microwave heating system, with HNO_3_:4 ml/H_2_O_2_:1 ml and heated to 100 °C over 5 min, held for 1 min and then heated to 200 °C over 5 min and held for 30 min as described in Sach et al. (2020).

Elemental analysis was conducted by inductively coupled plasma mass spectrometry (ICP-QQQ-MS; Agilent 8900x, USA) using collision reaction cell mode (reactive gas modes: H_2_ for Se at 7.0 ml/min, O_2_ for As at 30%, He for all remaining elements at 5.1 ml/min) for a suite of 58 elements using internal standards (Sc, Ge, Rh, In and Ir) for drift correction. ICP-MS operating parameters were: RF power 1550 W; plasma gas flow 15 L/min; carrier gas flow rate 1.0 L/min; sample solution uptake rate 0.4 ml/min. Fifteen biologically functional elements routinely used for human and wildlife health assessment (Sach et al., 2020) were selected for this study, including: Ca, Cu, Fe, K, Mg, Mn, Na, P, Se, Zn, As, Cd, Pb, U and V. The practical Limit of Detection was calculated as (LOD, 3*Std deviation x dilution) and is shown in Supplementary Information Table 1. The accuracy of the elemental analysis was verified by analysing the following Reference Materials (RM): Human Hair (GBW09101, Shanghai Institute of Nuclear Research, Academia Sinica, China) and in-house toenail reference material (BAPS 2014). The concentrations of all reference materials were found to be accurate within an acceptable percentage of the certified values for all elements studied (average % recovery = 97 ± 20%, see Supplementary Information Table 2).

## Results and discussion

### SEM

**Fig. 1:**
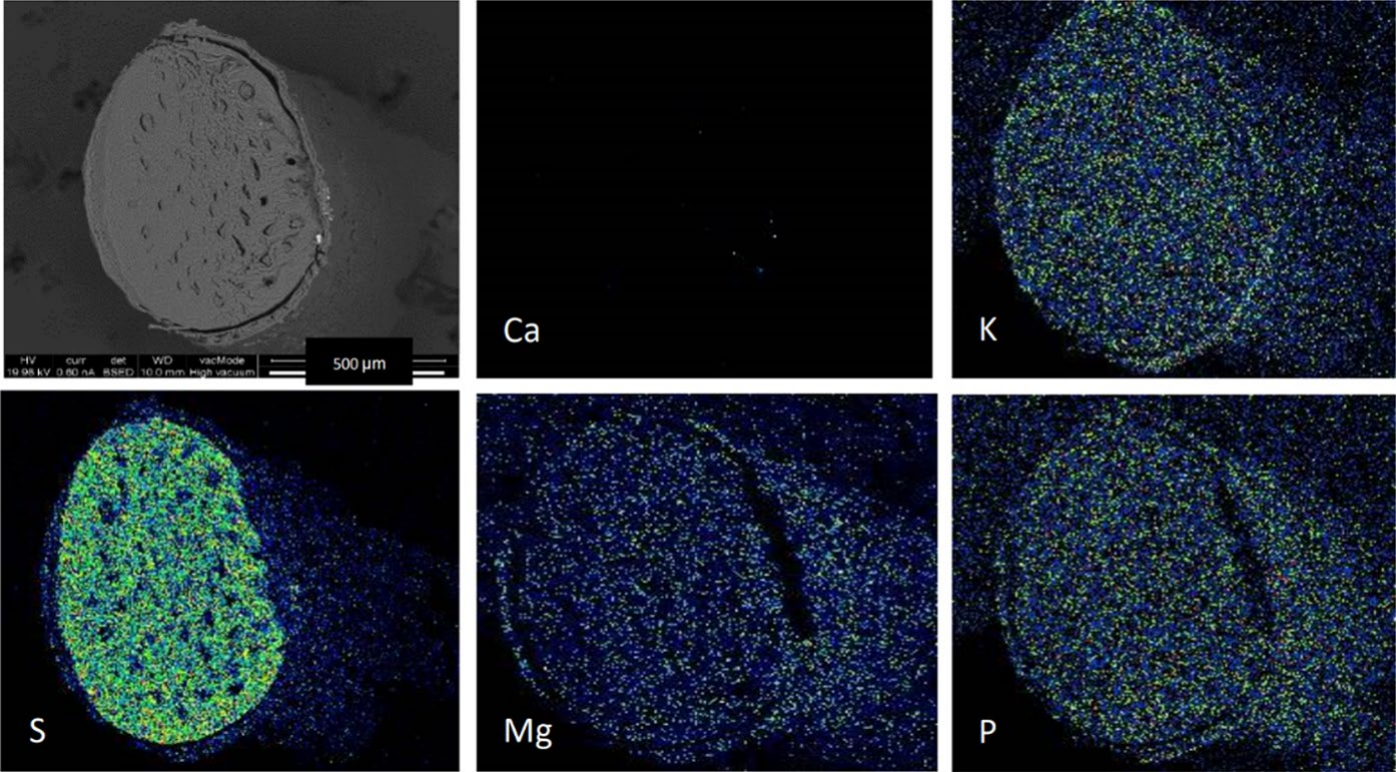
Energy-dispersive X-ray microanalysis (EDXA) elemental mapping of a cross-section of a wild female elephant tail hair. Black represents zero background through green, yellow and orange representing low to intermediate concentration to red or white, representing high concentration. The SEM image in the top left-hand corner shows the overall structure of the hair. Ca = calcium, K = potassium, S = sulphur, Mg = magnesium and P = phosphorus

**Fig. 2:**
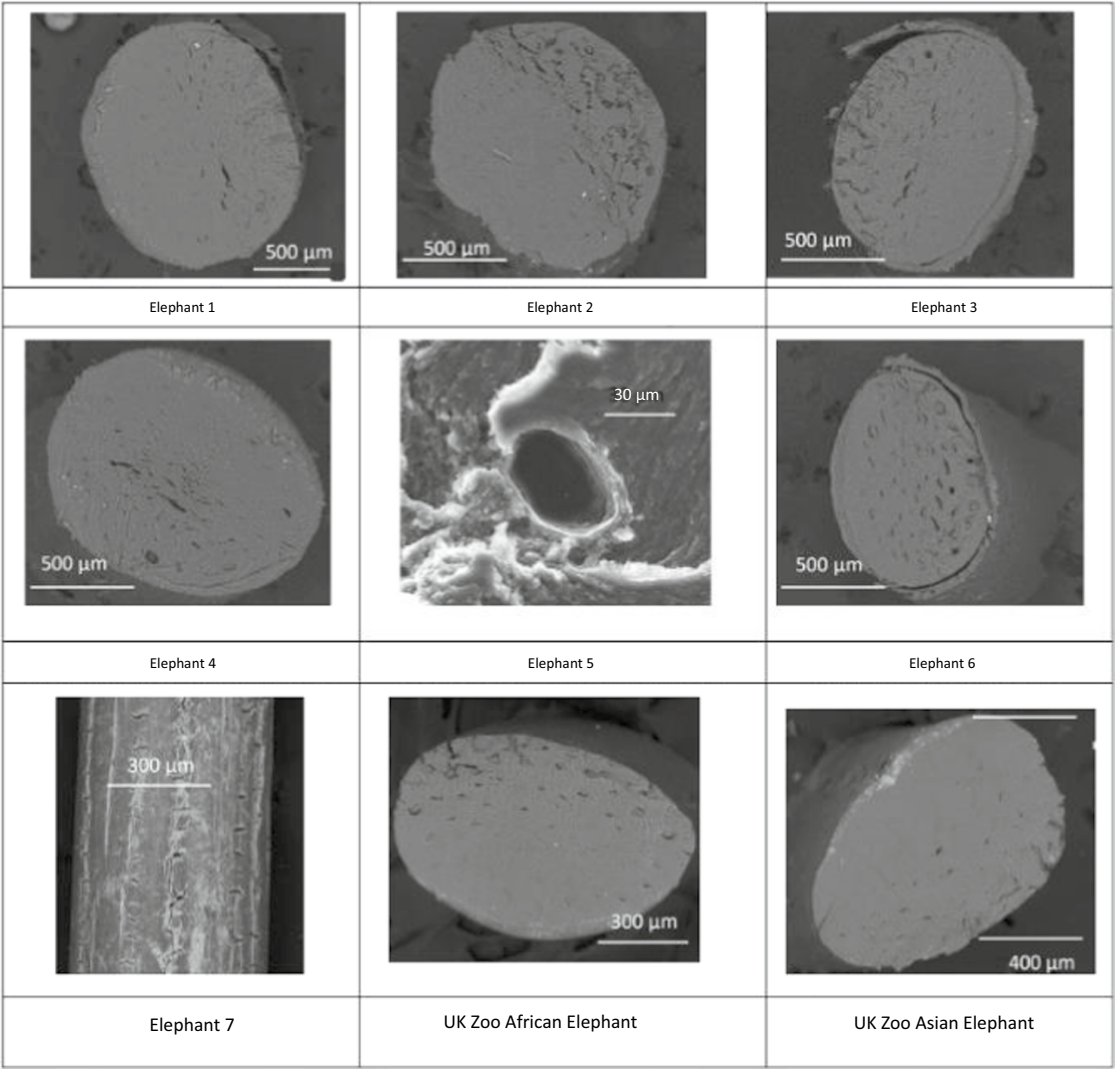
Scanning electron microscopy (SEM) images (cross-sectional and surface) of seven wild (elephants 1–7) and one Zoo African (*Loxodonta africana*) and one Zoo Asian (*Elephas maximus*) elephant tail hairs. Numbers are given to identify the elephants

Generally elemental distribution was uniform cross-sectionally, with clear delineation of K, S, Mg and P observed in Fig. 1 around the edge of the hair. Figure 2 shows SEM backscatter images of several zoo and wild elephants. This evaluation provided evidence that the cutting technique affected the illustration of the elemental distribution, and care must be taken to obtain a ‘clean cut’. As reported Yates et al. (2010), hairs show the presence of tubules, these may be infilled or empty and in African elephants are often found around the edges of the hair and not in the centre, hence care must be taken to avoid the tubules when sampling for LA-ICP-MS. This was especially visible in elephant 4 (Fig. 2). Secondly, the surface of the wild elephant hairs appeared to be cracked and rough, indicating potential for contamination that is impossible to remove, even with the extensive cleaning protocol, as seen in the image of the hair surface (Fig. 2).

**Fig. 3:**
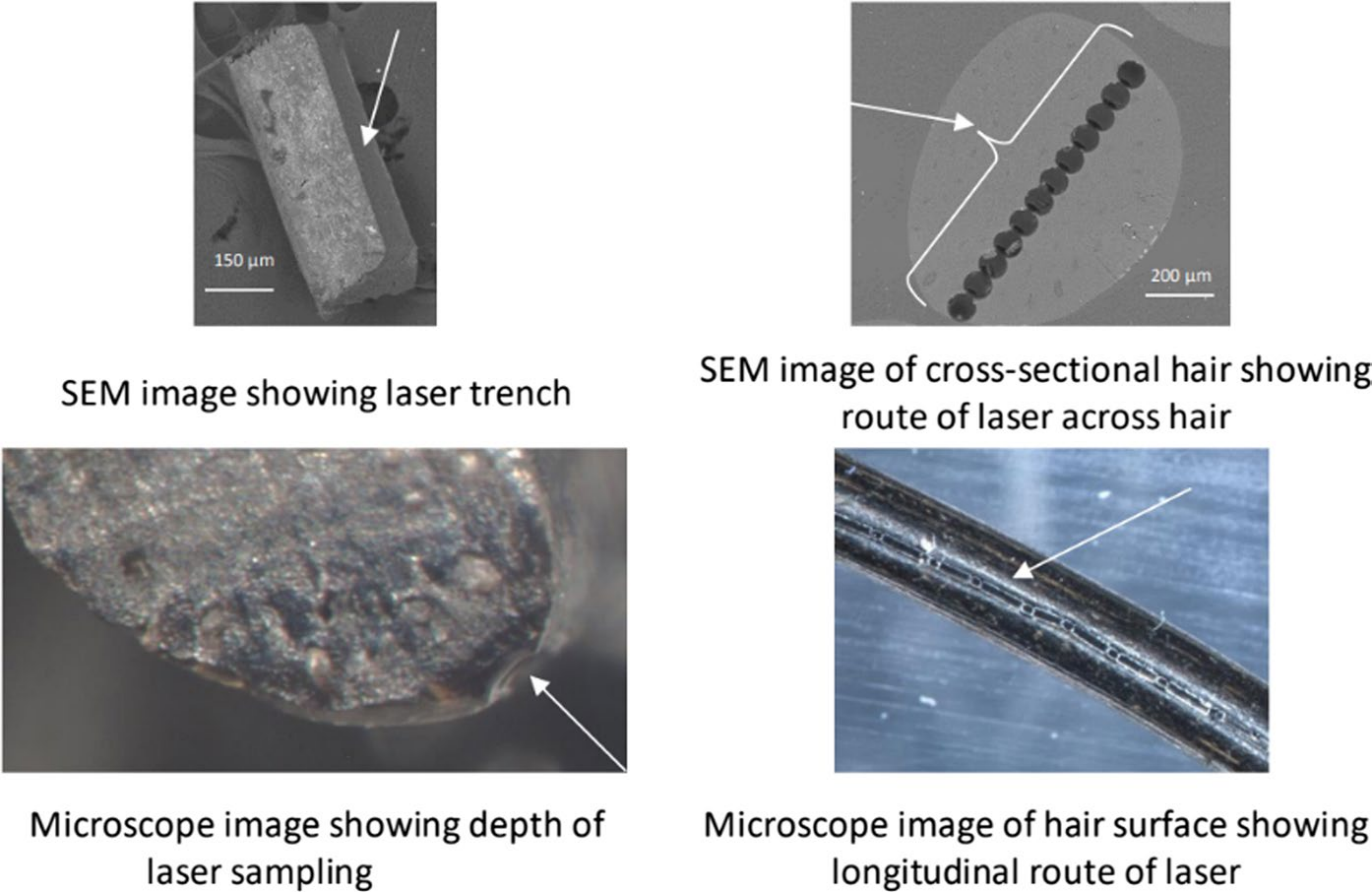
Images of tail hair taken after laser ablation was conducted to assess depth and pathway of laser sampling (cross-section spots and longitudinal trenches). SEM = Scanning Electron Microscopy

**Fig. 4:**
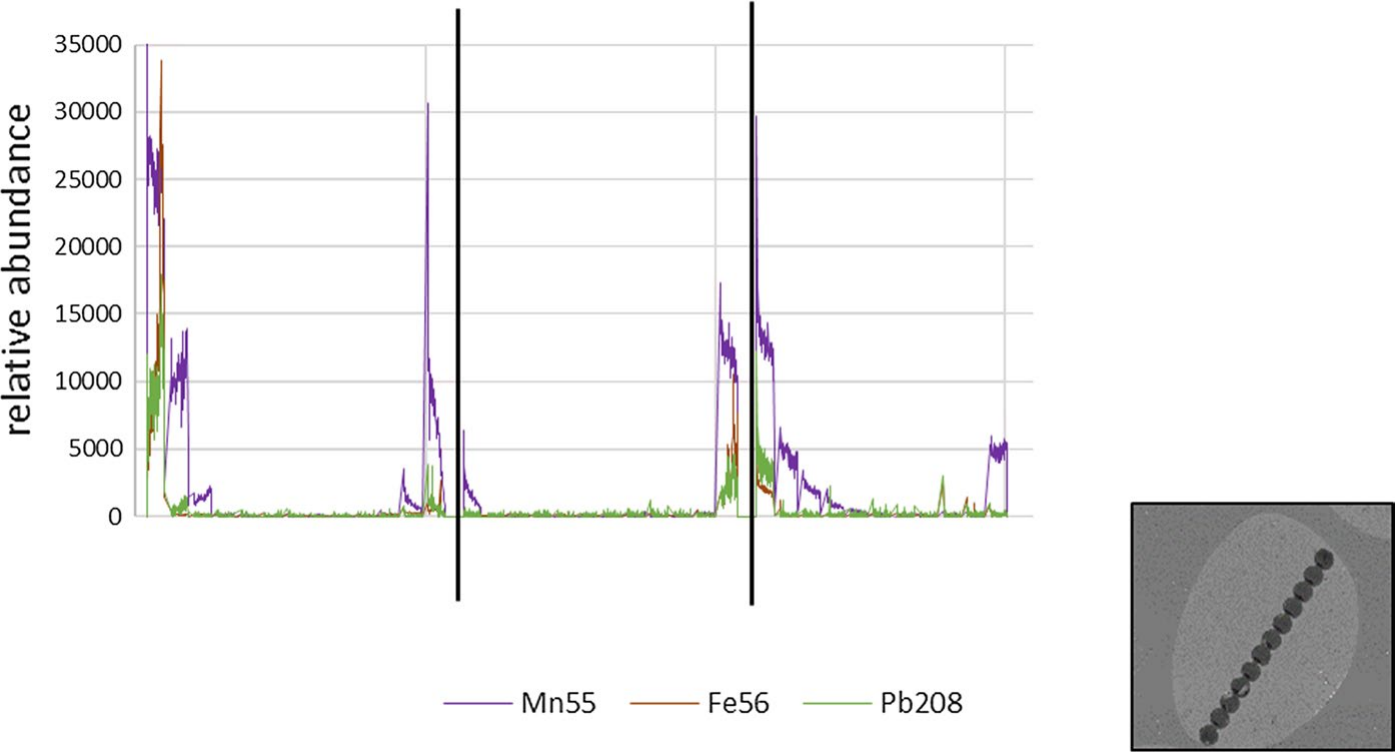
Cross-sectional laser ablation data across three cross-sectional pieces of African elephant tail hair. Elements present in greatest signal response as shown by the raw data output labelled on the y-axis as relative abundance are shown for Mn, Fe and Pb. Scanning electron microscopy image demonstrates the cross-sectional laser pathway across the hair

During method development, SEM and microscope images were taken of the lasered hair to review the path of the laser and determine appropriate sample depth as shown in Fig. 3. Additionally, cross-sectional examination of the hair was undertaken, to investigate the crusting around the edge of the hair, suspected to be an area of higher elemental concentrations, as seen on the previous SEM images (Figs. 1 and 2). The relative change in elements (Mn, Fe and Pb) can be seen across a cross section of elephant tail hair in Fig. 4. Areas of greatest elemental abundance/signal response were found around the edges of the hairs. Figures 1, 2, 3 and 4 suggest that the additional effort incurred from collecting spatial data through SEM imagery provides a constructive distribution picture to inform LA-ICP-MS sampling.

### Laser ablation and ICP-MS results

**Fig. 5:**
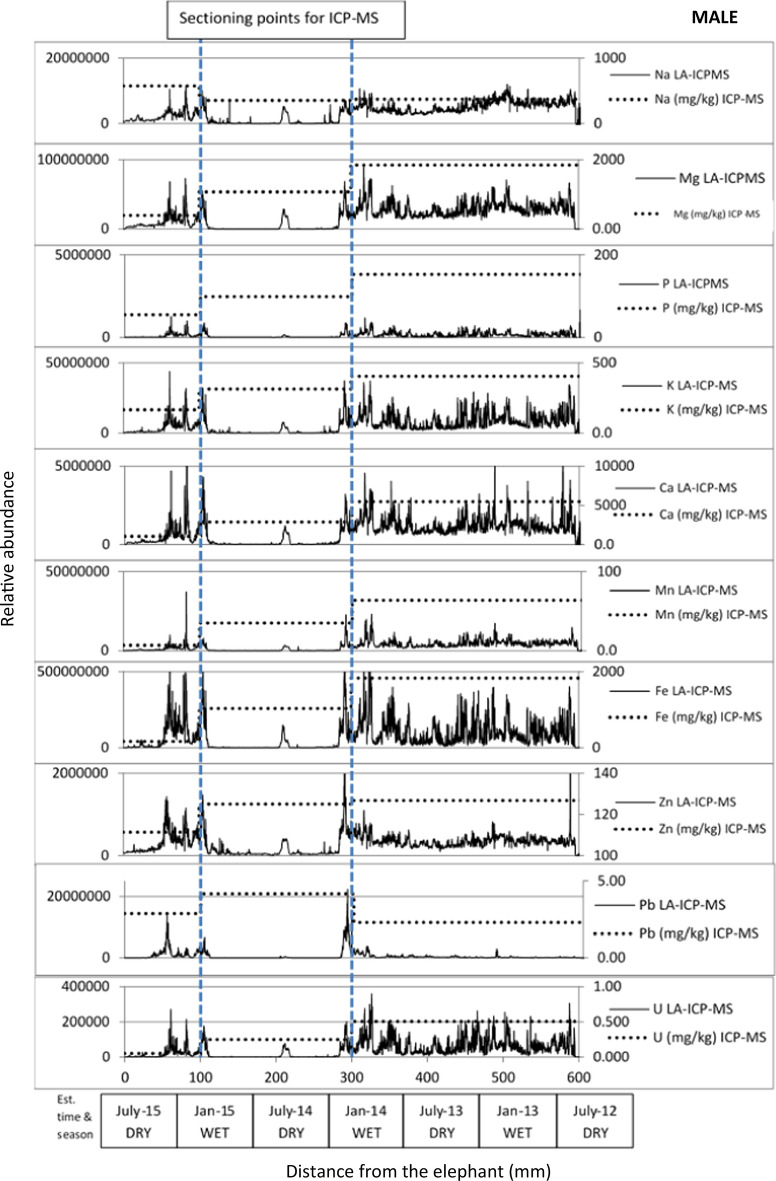

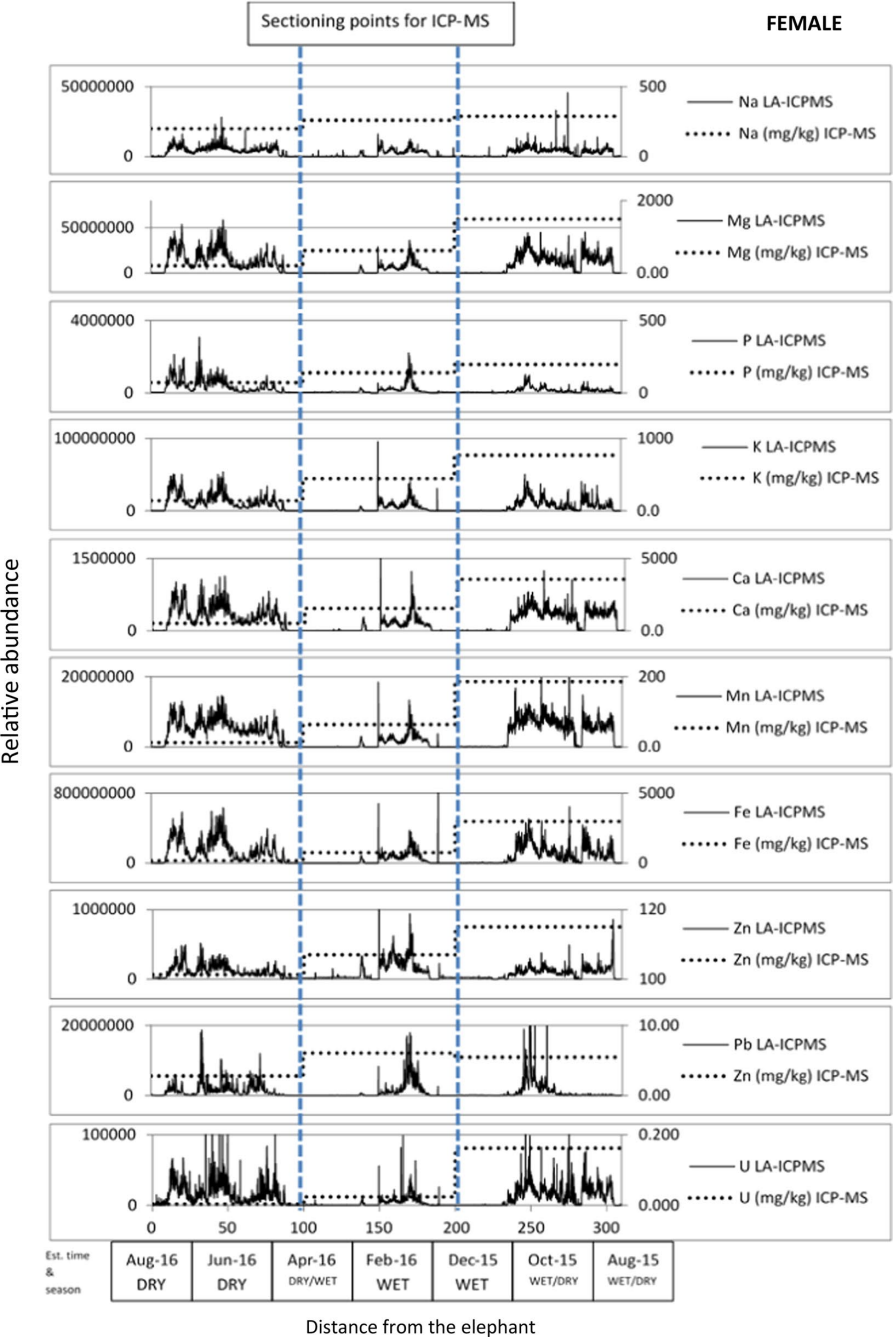
Laser ablation ICP-MS data (y-axis = relative abundance) for two wild African elephants, male and female, against estimated time and season data (x-axis). Distance from the elephant (mm), 0 = base of hair, attached to elephant tail. These hairs were sectioned for ICP-MS analysis as demonstrated by the blue dashed lines. ICP-MS analysis data also shown on right-hand axis

Figure 5 demonstrates how LA-ICP-MS informed where to section the tail hairs depending upon elemental concentrations, within the minimum mass limitations for subsequent ICP-MS analysis. The results for ICP-MS analysis are detailed in Supplementary Information Table 2 and summarised in Fig. 5. As the date of sampling was known, an approximate growth rate, for the elephant hair based on published averages—0.56 mm/day (± 0.11) in males and 0.81 mm/day (± 0.13) in females (Cerling et al., 2004; Wittemyer et al., 2009) was used to estimate the season from which the sample originated; growth rate must always be considered as an estimation.

In general, mineral/micronutrient and PTE levels increased down the hair as demonstrated by the solution chemistry by ICP-MS, with the male having higher overall mineral and PTE levels between Jan 2013 and Jan 2014, compared to Jan 2014–2015. In the female, the hair was shorter (about half the length) and mineral and PTE levels were higher between April 2016 to August 2016 compared to December 2015 to April 2016. The relationship between LA-ICP-MS data and ICP-MS solution data exhibited similar elemental chemistry for the male elephant for Mg, P, Ca, Fe and V and Mg, Na and Mn for the female elephant.

The LA-ICP-MS data for the sample from the female showed variation between the wet and dry seasons with relatively elevated levels of all elements investigated in the 2016 dry season, compared to the 2015/16 wet season, with an indication that this trend was mirrored in the 2015 dry season. Where possible, such data coupled with movement data could better inform the consequences of animals spending more/less time in areas with elevated geochemistry due to geogenic or anthropogenic activities e.g., mine tailings, and thus the elephants could be consuming increased levels of minerals or PTEs.

Combining the information in Figs. 1, 2, 3, 4 and 5 provided confidence that LA-ICP-MS of the hairs was conducted at an appropriate depth. This ensured inclusion of the high mineral crusting around the edge of the hair that was likely to show the greatest elemental variance down the hair, whilst avoiding the cracked outside, potentially contaminated surface of the hair. No substantial tubules were seen on SEM images of these elephants, giving assurance that the laser was targeted appropriately down the hair avoiding these tubules. It was suspected that elemental levels in tubules would be vastly reduced, as these are essentially air-filled.

## Conclusion

The methodology used here informed how the exposure to contrasting environmental geochemistry and other environmental factors can be reflected in the elemental profile down an elephant hair representing exposure over a period of time, tested using example tail hairs from wild African elephants commonly known to move between highly differing geochemistry settings – wild ranges often highly weathered with mineral deficient food sources to enriched mine tailings. LA-ICP-MS is a powerful tool to provide rapid time profiles for elemental chemistry that may reflect changes in exposure to environmental geochemistry, including seasonal changes in dietary intake. The use of SEM would not necessarily be required for routine analyses, but was highly useful in optimising the correct approach for LA-ICP-MS. Solution chemistry by ICP-MS provided quantitative data which may be further interest to reflect health status. However, LA-ICP-MS provides sufficient and rapid qualitative data to reflect more detailed changes over time in the geochemical exposure or dietary intake of animals or elephants in this case, whereas the solution chemistry approach will likely average data representing a time period on the tail hair. The power of inference for LA-ICP-MS data could be improved by generating quantitative data; this will require the development of new matrix matched reference standards for calibration or the validation of currently available non-matrix matched standards.

Future applications for LA-ICP-MS could inform environmental exposure and thus movement patterns of individual or populations of elephants, potentially indicating when elephants are spending time in areas of higher mineral provision, for example in mining areas or other anthropogenic activities or areas of crop raiding. Elevated soil mineral and PTE levels, caused from differences in local environmental geochemistry, may contribute towards increased mineral/micronutrient or PTE levels consumed via plants, soil and water by the elephant. Elephants adapt their movements to meet their resource needs, bringing them at times into conflict with humans (Enukwa, 2017). Approximately 70% of the range of African elephants is outside of protected areas, making human-elephant conflict (HEC) almost inevitable (Blanc et al., 2007; Wall et al., 2021). Understanding the multifactorial drivers of elephant movement, of which one is mineral provision reflected in the analysis of tail hair, is essential for land-use planning and management of elephant populations (Sach et al., 2019). This is required to understand the causes of HEC, and when attempting to alter elephant movement patterns or behaviours to reduce these conflicts (Grainger et al., 2005; Osborn, 2004; Stokke & Du Toit, 2002). Development of this methodology could inform conservation managers on elephant movement patterns and future conservation management decisions for the species. Such data may for example, be useful to inform changes in geochemistry exposure and subsequent influence on elephant movement over annual or decadal changes in the environment that may result from changes in land-use management or climate.

## Supplementary Information

Below is the link to the electronic supplementary material.

Supplementary file1 (XLSX 29 kb)

## Data Availability

Data available in supplementary information.

## References

[CR1] Armelin M, Avila R, Piasentin R (2003). Effect of chelated mineral supplementation on the absorption of Cu, Fe, K, Mn and Zn in horse hair. Journal of Radioanalytical and Neuclear Chemistry.

[CR3] Bencko V (1995). Use of human hair as a biomarker in the assessment of exposure to pollutants in occupational and environmental settings. Toxicology.

[CR4] Blanc, J. J., Barnes, R. F. W., Craig, G. C., Dublin, H. T., Thouless, C. R., Douglas-Hamilton, I. and Hart, J. A. (2007) African elephant status report 2007: an update from the African elephant database, African elephant status report 2007: An update from the African elephant database. 10.2305/iucn.ch.2007.ssc-op.33.en.

[CR6] Button M, Jenkin G, Harrington C, Watts M (2009). Human toenails as a biomarker of exposure to elevated environmental arsenic. Journal of Environmental Monitoring.

[CR7] Cerling TE, Passey BH, Ayliffe LK, Cook CS, Ehleringer JR, Harris JM, Dhidha MB, Kasiki SM (2004). Orphans’ tales: Seasonal dietary changes in elephants from Tsavo National Park Kenya. Palaeogeography, Palaeoclimatology, Palaeoecology.

[CR8] Chiyo PI, Cochrane EP, Naughton L, Basuta GI (2005). Temporal patterns of crop raiding by elephants: A response to changes in forage quality or crop availability?. Journal of African Ecology.

[CR9] Combs D (1987). Hair analysis as an indicator or mineral status of livestock. Journal of Animal Science.

[CR10] Enukwa EH (2017). Human-Elephant confilict mitigation methods: A review of effectiveness and sustainability. Journal of Wildlife and Biodiversity.

[CR12] Grainger M, Van Aarde R, Whyte I (2005). Landscape heterogeneity and the use of space by elephants in the Kruger National Park, South Africa. African Journal of Ecology.

[CR13] Hausman LA (1920). Structural characteristics of the hair of mammals. American Naturalist.

[CR14] Hoover RR, Jones CM (2013). Effect of laser ablation depth in otolith life history scans. Marine Ecology Progress Series.

[CR15] Hurst R, Siyame EWP, Young SD, Chilimba ADC, Joy EJM, Black CR, Ander EL, Watts MJ, Chilima B, Gondwe J, Kang’ombe D, Stein AJ, Fairweather-tait SJ, Gibson RS, Kalimbira AA, Broadley MR, Kang D, Kang’ombe D, Stein AJ, Fairweather-tait SJ, Gibson RS, Kalimbira AA, Broadley MR (2013). Soil-type influences human selenium status and underlies widespread selenium deficiency risks in Malawi. Scientific Reports.

[CR17] Joy E, Broadley M, Young S, Black C, Chilimba A, Ander L, Barlow T, Watts M (2015). Soil type influences crop mineral composition in Malawi. Science of the Total Environment, the.

[CR19] Middleton DRS, Watts MJ, Hamilton EM, Fletcher T, Leonardi GS, Close RM, Exley KS, Crabbe H, Polya DA (2016). ‘Environmental science processes & impacts supplies : Toenail, hair and drinking water. Environmental Science: Processes & Impacts Royal Society of Chemistry.

[CR20] Milton DA, Chenery SR (2005). ‘Movement patterns of barramundi Lates calcarifer, inferred from 87Sr/86Sr and Sr/Ca ratios in otoliths, indicating non-participation in spawning. Marine Ecology Progress Series.

[CR21] Osborn F (2004). The concept of home range in relation to elephants in Africa. Pachyderm.

[CR25] Sach F, Dierenfeld ES, Langley-Evans SC (2020). Potential bio-indicators for assessment of mineral status in elephants. Science and Reports.

[CR26] Sach F, Yon L, Henley M, Bedetti A, Buss P, de Boer WF, Dierenfeld E, Gardner A, Langley-Evans S, Hamilton E, Lark RM, Prins HHT, Swemmer AM, Watts M (2020). Spatial geochemistry influences the home range of elephants. Science of the Total Environment.

[CR27] Sach F, Dierenfeld E, Langley-Evans S, Watts M, Yon L (2019). African savanna elephants (Loxodonta africana) as an example of a mega herbivore making movement choices based on nutritional needs. Peer Journal.

[CR28] Sela H, Karpas Z, Zoriy M, Pickhardt C, Becker JS (2007). Biomonitoring of hair samples by laser ablation inductively coupled plasma mass spectrometry (LA-ICP-MS). International Journal of Mass Spectrometry.

[CR29] Standard operating procedures for capture transport and maintenance in holding facilities of wildlife (2017). *South African National Parks*.

[CR30] Stokke S, Du Toit JT (2002). Sexual segregation in habitat use by elephants in Chobe National Park, Botswana. African Journal of Ecology.

[CR32] Wall J, Wittemyer G, Klinkenberg B, LeMay V, Blake S, Strindberg S, Henley M, Vollrath F, Maisels F, Ferwerda J, Douglas-Hamilton I (2021). Human footprint and protected areas shape elephant range across Africa. Current Biology.

[CR33] Wittemyer G, Cerling TE, Douglas-Hamilton I (2009). Establishing chronologies from isotopic profiles in serially collected animal tissues: An example using tail hairs from African elephants. Chemical Geology.

[CR34] Yates BC, Espinoza EO, Baker BW (2010). Forensic species identification of elephant (*Elephantidae*) and giraffe (*Giraffidae*) tail hair using light microscopy. Forensic Science, Medicine, and Pathology.

